# The effective constituent puerarin, from *Pueraria lobata*, inhibits the proliferation and inflammation of vascular smooth muscle in atherosclerosis through the miR-29b-3p/IGF1 pathway

**DOI:** 10.1080/13880209.2022.2099430

**Published:** 2022-12-20

**Authors:** Jianpeng Li, Yanan Li, Xiangke Yuan, Dengfeng Yao, Zongyue Gao, Zhaoyang Niu, Zheng Wang, Yue Zhang

**Affiliations:** a The First Clinical Medical College, Shandong University of Traditional Chinese Medicine, Jinan City, Shandong Province, China; b Department of Peripheral Vascular, Henan Provincial Hospital of Traditional Chinese Medicine (The Second Affiliated Hospital of Henan University of Traditional Chinese Medicine), Zhengzhou City, Henan Province, China; c Department of Nephrology, The First Affiliated Hospital of Henan University of Traditional Chinese Medicine, Zhengzhou City, Henan Province, China; d Department of Peripheral Vascular, Affiliated Hospital of Shandong University of Traditional Chinese Medicine (Shandong Provincial Hospital of Traditional Chinese Medicine), Jinan City, Shandong Province, China

**Keywords:** Inflammatory factors, hVSMC

## Abstract

**Context:**

Atherosclerosis (AS) is the main cause of cardiovascular and cerebrovascular diseases. *Pueraria lobata* (Willd.) Ohwi (Fabaceae) has a positive effect on improving these diseases.

**Objective:**

The *P. lobata* effect on the proliferation and inflammation of vascular smooth muscle in AS and the potential mechanism were investigated.

**Materials and methods:**

By feeding a high-fat diet to 8-week-old apolipoprotein E knockout mice, an atherosclerosis model was created. H&E and IHC staining were used to analyse the histopathology of mice. CCK-8, TUNEL, and scratch tests were used to detect cell proliferation, apoptosis, and migration after 24 h treatment, respectively. ELISA was performed to evaluate the level of IL-6 and IL-8. The target miRNA and its downstream target gene were screened by the bioinformatics method; RT-qPCR has conducted to analyse the expression of these genes.

**Results:**

In the aortic tissue and serum of AS mice, puerarin can lower the expression of α-SMA and the inflammatory proteins IL-6 and IL-8. Puerarin (200 M) decreased hVSMC proliferation, migration, and IL-6 and IL-8 secretion by more than half. The inhibitory impact of puerarin on hVSMC was decreased by overexpression of miR-29b-3p. IGF1 was miR-29b-3p's downstream target gene. IGF1 expression increased almost 3-fold in AS mice and hVSMC, but miR-29b-3p mimic inhibited it. The effect of miR-29b-3p on hVSMC was reversed when IGF1 was overexpressed.

**Discussion and conclusions:**

Puerarin inhibits the proliferation and inflammation of vascular smooth muscle in AS through the miR-29b-3p/IGF1 pathway. Puerarin may have a beneficial effect in the treatment of atherosclerosis and offer a novel therapy option.

## Introduction

Atherosclerosis (AS) is one of the most common arterial diseases related to changes in blood vessel wall composition and dyslipidemia. It is an important cause of most cardiovascular diseases, cerebral infarction, strokes, and peripheral vascular diseases, and seriously endangers the health of patients (Rahman and Woollard 2017; Kobiyama and Ley 2018). According to epidemiological investigations, cardiovascular diseases associated with AS are the largest cause of death worldwide, and ischaemic heart disease and stroke caused by AS are the biggest killers, causing more than 15 million deaths worldwide every year (Zhu et al. 2018). With the advent of an ageing global population, the prevalence of AS is also increasing rapidly, which seriously threatens the quality of human life and also brings an astonishing economic burden (Xu et al. 2018). The occurrence and development of AS is a very complex process, which includes various mechanisms such as cholesterol metabolism disorder, injury response, inflammatory response, and vascular smooth muscle proliferation (Chen et al. 2019; Libby et al. 2019; Genkel et al. 2020). Inflammation and cholesterol metabolism disorders are considered to be the main pathogenesis of AS (Kattoor et al. 2017; Wolf and Ley 2019). Investigating effective prevention and control targets of AS has become a hot research issue.

The main anti-AS drugs used in clinical practice are hypolipidemic drugs, antiplatelet agglutinating drugs, antioxidants, etc. (Zarate et al. 2016; Chistiakov et al. 2018). However, these drugs have varying degrees of limitations, for example, studies have found that although lipid-lowering drugs can reduce low-density cholesterol levels, there is still a high incidence of cardiovascular disease, and lipid-lowering drugs have a certain degree of damage to liver function, while antiplatelet drugs tend to cause bleeding (Vaidyanathan and Gopalakrishnan 2017; Gao et al. 2020). Recently, studies have found that some active constituents contained in many traditional Chinese medicines have a good prevention and treatment effect on AS (Wang et al. 2018). Therefore, it is important to determine the key constituents of traditional Chinese medicine for the therapy of AS and to seek the possible molecular mechanisms of the therapeutic effects.

*Pueraria lobata* is the dried roots of legumes *P. lobata* (Willd.) Ohwi (Fabaceae) and *Pueraria thomsonii* Benth (Fabaceae). It is often used clinically in gynecological, cardiovascular, cerebrovascular diseases, etc. (Wang et al. 2020). Modern pharmacological studies have shown that *P. lobata* can effectively improve cardiovascular and cerebrovascular diseases, it has the effects of lowering blood lipids and blood sugar, and it has a positive effect on AS and coronary heart disease, etc. (Zhou et al. 2014). The results of a randomised controlled trial showed that *Salvia miltiorrhiza* Bunge (Labiatae) and *P. lobata* treatment in coronary patients was well tolerated and can effectively improve vascular structure and function in patients with coronary heart disease (Tam et al. 2009). The main active constituents of *P. lobata* are isoflavone compounds, mainly including daidzein, puerarin, and puerarin-7-xyloside (Cui et al. 2018; Wang et al. 2019a). In recent years, it has been reported that the isoflavones in *P. lobata* have the effects of dilating blood vessels and coronary arteries, improving microcirculation, anti-arrhythmia, lowering blood pressure and blood lipids (Chen et al. 2018; Zhang et al. 2018). Their potential for prevention and therapy of cardiovascular and cerebrovascular diseases has gradually been discovered and developed, and they are expected to become a new force in the prevention and treatment of AS, but the specific mechanism is still unclear.

Network pharmacology is a research method proposed by Hopkins et al. to explore the relationship between drugs and diseases (Hopkins 2008). It is an emerging discipline that integrates molecular biology, pharmacology and various network database platforms based on the background of big data. Network pharmacology aims to establish a network of “constituents-targets-pathways-disease” from multiple dimensions through screening, so as to explain the molecular mechanism of Chinese medicine prescriptions or constituents acting on diseases more intuitively (Luo et al. 2020). In this study, the effective constituents and targets of *P. lobata* in treating AS were excavated and analysed by virtue of the method of network pharmacology, and the effect of a key component of *P. lobata* on AS was verified *in vitro* and *in vivo*. In addition, we carried out GO function analysis on the targets of the effective constituents of *P. lobata*, and mechanisms of the selected miRNA and its downstream targets in AS were also discussed.

## Materials and methods

### Screening the active constituents of Pueraria lobata

The chemical constituents of *P. lobata* root were obtained through the database named Traditional Chinese Medicine Systems Pharmacology Database and Analysis Platform (TCMSP) (Appendix). OB (Oral Bioavailability) ≥ 20% and DL (Drug-Like) ≥ 0.1 were used as a filter to screen the active constituents of *P. lobata*.

### Prediction of the targets through the comparative toxicogenomics database (CTD)

The CTD database (Appendix) was chosen to screen out the targets of the active constituents of *P. lobata*, then the target genes related to AS were screened out with the keyword “atherosclerosis” in the CTD database. The online Venn diagram (Appendix) was used to deal with the selected targets to obtain the intersection genes.

### Gene ontology (GO) analysis

Targets of puerarin for AS treatment were input into the DAVID database (Appendix), the species selection was “Homo sapiens,” then we performed the analysis. Finally, the target miRNAs were screened and sorted in ascending order of the *P*-value (*P* ≤ 0.01).

### Prediction and screening of target genes of miRNA

Several databases were applied to predict the target genes of miR-29b-3p, including Target Scan, miRDB, Starbase, and RNA-society. At the same time, the PaGenBase database was used to screen the target genes enriched in smooth muscle (Appendix).

### Cell culture and as cell model establishment

Human vascular smooth muscle cells (hVSMC) were obtained from the Shanghai Cell Bank of the Chinese Academy of Sciences. High glucose DMEM medium (Gibco, USA) added with 10% FBS (Gibco, USA) and 1% dual-antibodies (Ncmbio, China) was used for cell culture, and the cultural environment was 37 °C with 95% air and 5% CO_2_. After resuscitation, the cells needed to be cultured to the third generation before they could be used in follow-up experiments. The AS cell model was established by 20 μg/mL ox-LDL induction of hVSMC for 24 h.

### Cell transfection

hVSMC was incubated in a 24-well plate in advance until the cell confluence was up to about 40%. Commercial RNA transfection reagent Entranster^TM^-R4000 (Engreen Biosystem, China) was applied to transfect miRNA mimics or sh-RNA. MiR-29b-3p mimics, NC mimics, pcDNA-IGF1 and pcDNA-NC were synthesised by Sangon Biotech. Specifically, the miRNA mimics or pcDNA were diluted in a serum-free medium, then mixed with the diluted transfection reagent, and added to the well. After 48 h, subsequent experiments could be carried out.

### Detection of cell proliferation

The ox-LDL-induced hVSMC cells with a confluence of more than 80% were digested and cultured in a 96-well plate. When the cells were tightly adherent to the wall, they were split into five groups: blank group, 50, 100, and 200 μM puerarin groups, and fluvastatin (100 μM) group. Each group contained 6 parallel wells. The puerarin was obtained from Macklin Biochemical Co., Ltd. (Shanghai, China), and the positive medicine fluvastatin was purchased from Bidepharm (Shanghai, China). Puerarin was prepared as 100 mM mother liquor with sterile water, fluvastatin was dissolved with DMSO and prepared as 50 mM mother liquor, then they were diluted to the working concentration with DMEM medium. Next, each group was added with the corresponding drugs, and cells were cultured for 24 h, then 10 μL/well of CCK-8 solution (Beyotime, Nanjing, China) was injected and reacted at 37 °C for 2–4 h. In the end, the absorbance value at OD_450 nm_ was detected by the microplate reader (Biotek Synergy H1, USA).

### Detection of cell apoptosis

The TUNEL Apoptosis Assay Kit (Beyotime, Nanjing, China) was applied to detect the apoptosis of ox-LDL-induced hVSMC after puerarin treatment. In brief, cells were first treated with low, medium, high concentrations of puerarin or fluvastatin, then fixed with 4% fixative paraformaldehyde and permeabilized with 0.3% Triton X-100. After that, 50 μL of the newly configured TUNEL detection solution was added to the sample and reacted in the dark at 37 °C for 30 min. After rinsing, the cell slides were sealed with a fluorescence decay-resistant reagent containing DAPI (Solarbio, Beijing, China). The cell apoptosis was observed and recorded under a laser confocal microscope (Nikon A1, Japan).

### Detection of cell migration ability

The scratch test was performed to measure the migration capacity of hVSMC after puerarin treatment. ox-LDL-induced hVSMC were inoculated in a 24-well plate and cultured to a confluence of more than 90%. The cells were divided into 5 groups and a straight line was drawn on the monolayer cells with a sterile needle. After rinsing with PBS, 50, 100, 200 μM puerarin and 100 μM fluvastatin diluted with DMEM medium were added to the 4 experimental groups, an equal amount of DMEM medium was added to the blank group and continued to incubate for 24 h. Finally, the wound healing was observed with the microscope and quantified with the image acquisition system.

### Establishment of mouse atherosclerosis model and puerarin treatment

In this experiment, a total of 25 apolipoprotein E knockout mice (ApoE^−/−^ mice) 8-weeks-old were used to construct an atherosclerosis model (*n* = 5). The mice were provided by the Henan University of Traditional Chinese Medicine. The experimental protocol has been approved by the Animal Ethics Committee of Henan University of Traditional Chinese Medicine (HUTCM-20201215). All mice could eat and drink freely, the environment temperature was controlled at 22 ± 2 °C, and the humidity was about 50–60% with a 12 h light/dark cycle. After adaptive feeding for one week, the mice were modelled. ApoE^−/−^ mice were fed with high-fat diet (15% lard, 2% cholesterol, 0.2% sodium cholate). After 9 weeks of modelling, the blood lipid constituents such as TG, TC, LDL-C, and HDL-C were tested to determine whether the modelling was successful.

After the AS mice were induced successfully, the remaining 25 mice in the model group were randomly divided into 5 groups: the model control, fluvastatin (4 mg/kg/d), low-dose (50 μM/kg/d), medium-dose (100 μM/kg/d), and high-dose puerarin (200 μM/kg/d) groups. All groups were administered intragastrically; the control group was given 0.9% saline once a day for 4 weeks.

### Detection of the inflammatory factor expression level

ELISA method was used to assess the level of inflammatory cytokines IL-6 and IL-8 in mice serum or supernatant of the ox-LDL-induced hVSMC. Human IL-6, IL-8 detection kit (ELISA), and mouse IL-6 detection kit (ELISA) were purchased from Biolab (Beijing, China), and mouse IL-8 detection kit (ELISA) was purchased from Westang (Shanghai, China). The experiment was carried out in accordance with the instructions, and the absorbance of OD_490nm_ was measured with the microplate reader. The concentration of IL-6 and IL-8 was determined through the drawn standard curve.

### Hematoxylin-eosin (HE) staining, oil red O staining, and immunohistochemistry (IHC)

All mice were anaesthetised with sodium pentobarbital and then the blood was collected and the mice sacrificed by cervical dislocation. The mice were then dissected and two sections of 2–3 mm slices were cut from the aortic roots. One section was fixed in 10% neutral formaldehyde, then the fixed slices were embedded in paraffin and cut into sections with a thickness of 4 μm with a microtome (Leica, Germany). The other section was embedded with OTC and sectioned with a freezing microtome (Leica, Germany) to a thickness of 8 μm and stored at −20 °C.

HE staining: Aortic tissue sections of each group were deparaffinized with xylene, hydrated with gradient ethanol, and then underwent conventional HE staining. Finally, the film was sealed with neutral resin, observed, and photographed under the microscope.

Oil Red O staining: Frozen sections were dried at room temperature for 1 h, then rinsed with 60% isopropyl alcohol for 30 s. The sections were stained with Oil Red O solution (Sigma-Aldrich, USA) for 20 min, and differentiated with 70% ethanol until normal tissues turned white. Afterward, the sections were rinsed with PBS, dried, and observed under a microscope.

IHC: According to the conventional steps of IHC, we performed deparaffinization, hydration, high-pressure repair in sequence, and then the slices were incubated at 4 °C overnight with anti-α-smooth muscle actin (α-SMA) polyclonal antibody (1:1000 dilution, Santa Cruz, CA, USA) diluted with bovine serum. The next day, the slices were placed in the diluted secondary antibody (goat anti-mouse, 1:2000 dilution, Santa Cruz, CA, USA) and incubated for 2 h at 25 °C. DAB reagent (Sangon Biotech, China) was applied to visualise the localisation of the HRP-conjugated antibody. Finally, the slices were sealed with neutral resin and recorded under a microscope for analysis.

### Real-time quantitative PCR (RT-qPCR)

RT-qPCR was used to detect the relative level of possible target miRNAs and target genes in mouse tissues and hVSMC cells. First, the total RNA of mouse aortic tissue or hVSMC cells was extracted by TRIzol reagent (Thermo Fisher, USA), and the quality and concentration of the extracted RNA were detected by UV spectrophotometry. Then, the 5 × TranScript All-in-One SuperMix for qPCR kit (TransGen Biotech, China) was applied to synthesise cDNA according to the instructions, and the obtained cDNA was stored at −80 °C. Finally, the TransScript Tip Green qPCR SuperMix kit (TransGen Biotech, China) was used. After the qPCR reaction, the 2^-ΔΔCt^ method was applied to analyse the relative expression levels of the tested genes. The primers used in this research are synthesised by Shanghai Sangon Biotech Co., Ltd.

### Western blot (WB)

RIPA lysis (Pierce) was employed for extracting proteins. Proteins were separated by sodium dodecyl sulfate-polyacrylamide gel electrophoresis (SDS-PAGE) and transferred on PVDF membranes. Following Bloced by skim milk, the membranes were immunoblotted with primary antibodies including IGF1 (1.5 µg/mL, ab106836, Abcam) and β-actin (1 µg/mL, ab8226, Abcam). Post washing by Tris-buffer with Tween 20, secondary antibody goat anti-rabbit IgG- horseradish peroxidase (HRP) conjugate (0.5 µg/mL, ab6721, Abcam) was filled in and incubated for 1 hour. Subsequently, an ECL Western Blotting Substrate Kit (TransGen Biotech, China) was applied to visualising the protein bands.

### Statistical analysis

The SPSS23.0 statistical software was used for statistical analysis, and the data were shown as mean ± standard deviation (x ± s), and statistical two-tailed Student’s *t*-test was applied to compare two conditions, whereas a one-way ANOVA statistical test was used to compare multiple conditions. *P* < 0.05 means the difference is statistically significant.

## Results

### Puerarin is an effective constituent of *Pueraria lobata* in the treatment of atherosclerosis

**Figure 1.**
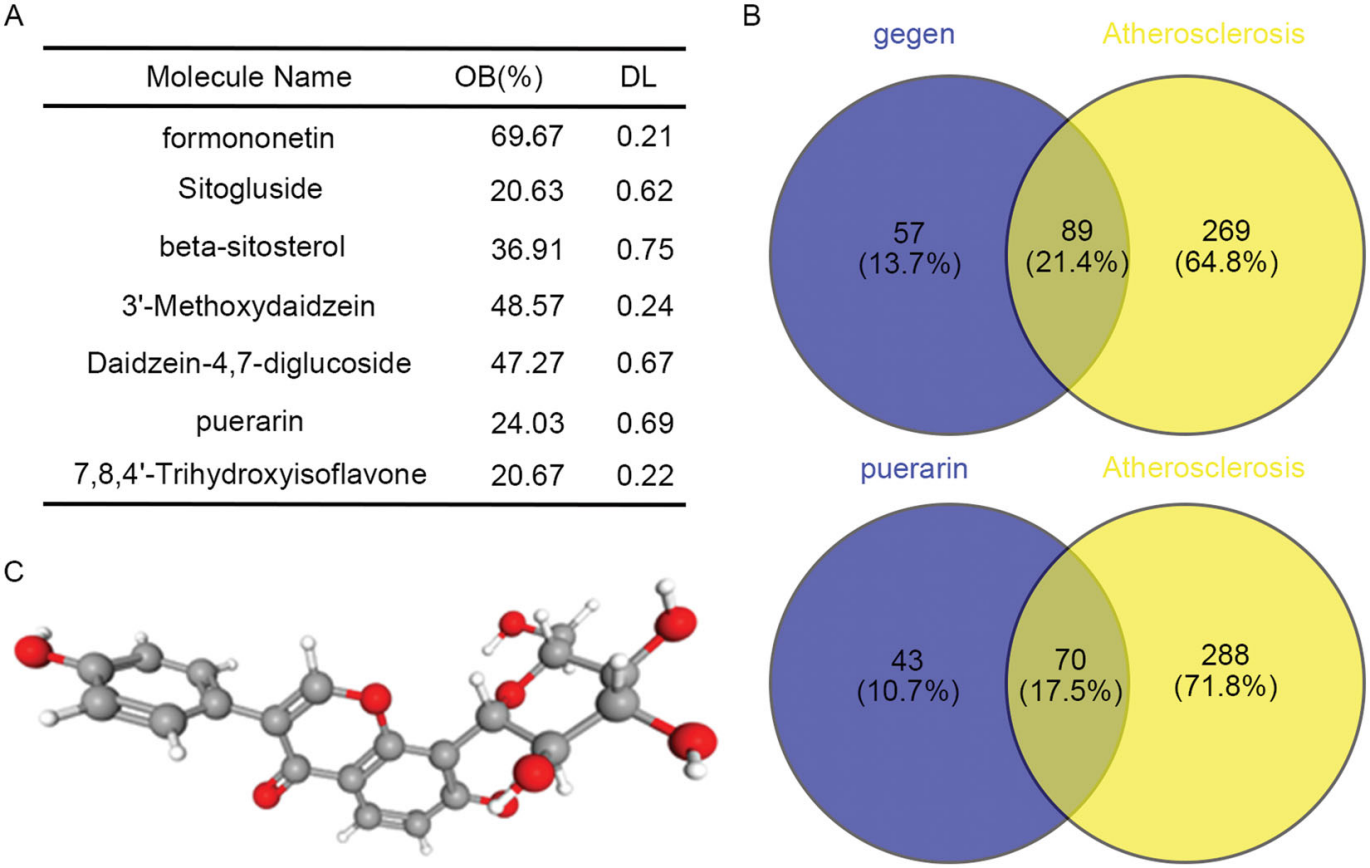
Puerarin is the effective constituent of *P. lobata* in the treatment of atherosclerosis. (A) The effective constituents of *P. lobata* were screened by TCMSP according to OB ≧ 20% and DL ≧ 0.1. (B) Venn diagram of the targets of the screened 7 effective constituents of *P. lobata* and the puerarin intersecting with the targets related to atherosclerosis. (C) The molecular structure diagram of puerarin was obtained from the PubChem database.

A total of 18 constituents of *P. lobata* were retrieved by the TCMSP database. According to the requirements of easy absorption and utilisation of drugs, OB ≥ 20% and DL ≥ 0.1 were set as the screening conditions, and a total of 7 active constituents of *P. lobata* were screened out (Figure 1A). According to the prediction of the CTD database, there were 146 targets of the 7 active constituents of *P. lobata*. By intersecting the targets related to AS, a total of 89 intersections were obtained (Figure 1B, Up). Puerarin had 113 targets, of which 70 targets were related to AS (Figure 1B, Down). Therefore, we speculated that puerarin was the effective constituent of *P. lobata* in the treatment of AS. Moreover, we obtained the molecular structure diagram of puerarin from the PubChem database (Figure 1C), and the molecular formula of puerarin is C_21_H_20_O_9_.

### Puerarin can alleviate atherosclerosis in mice

**Figure 2.**
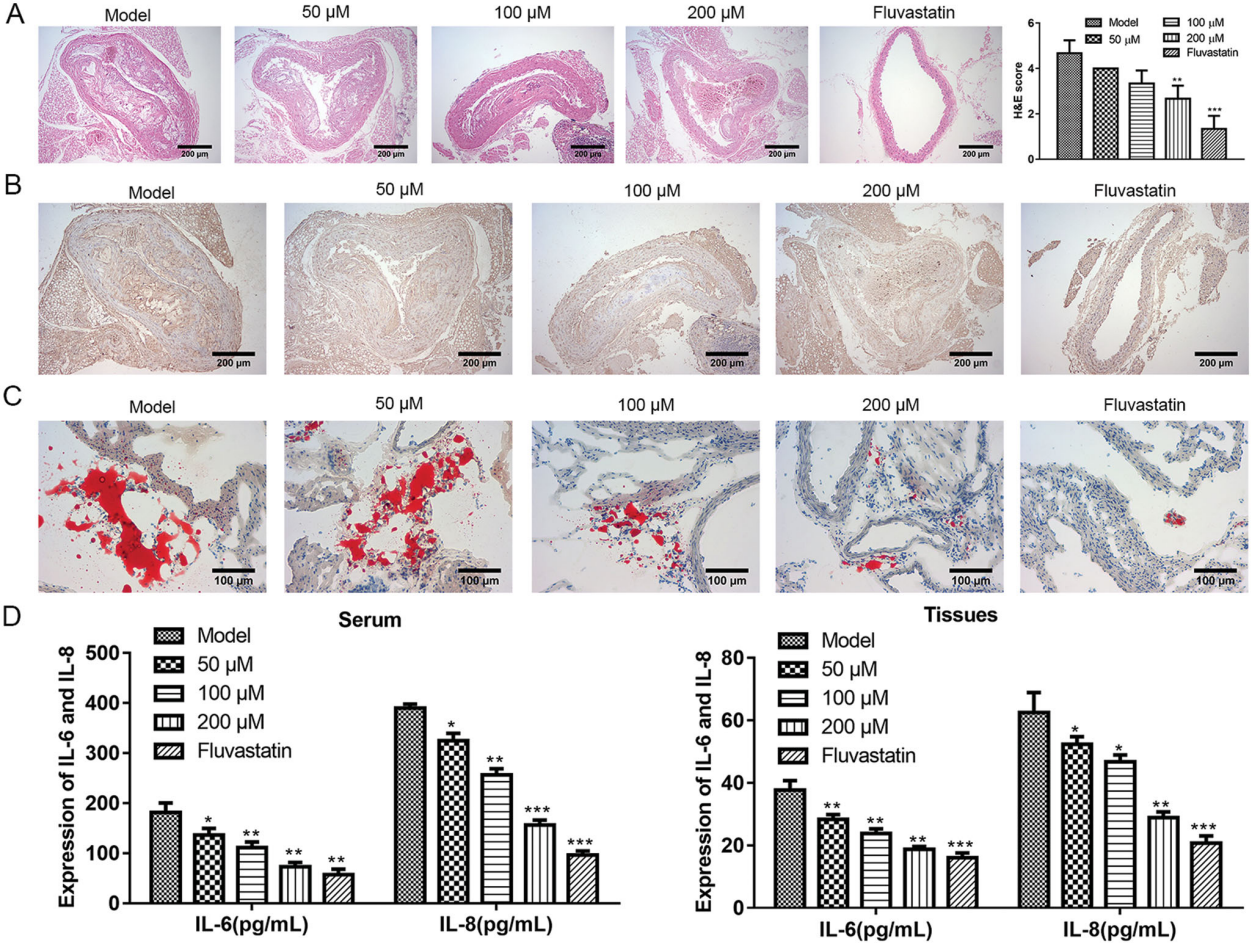
The therapeutic effect of puerarin on atherosclerosis model mice. (A) HE staining and HE scores of aortic tissue of mice in the model group, 50, 100, 200 μM puerarin-treated groups and fluvastatin-treated group. Scale bar =100 μm. (B) IHC method was applied to examine the expression level of α-SMA in the aorta of mice in model group, 50, 100, 200 μM puerarin treatment groups and fluvastatin treatment group. Scale bar =100 μm. (C) Oil Red O staining of aortic tissues in the model group, 50, 100, 200 μM puerarin-treated groups and fluvastatin-treated group. Scale bar =100 μm. (D) The expression level of pro-inflammatory cytokines IL-6 and IL-8 in the serum (left) and tissues (right) of the five groups of mice. *P < 0.05, **P < 0.01, ***P < 0.001.

We want to know whether puerarin really has a relieving effect on AS in animals. To this end, we first established the mouse model of AS through a high-fat diet, and fed the model mice with different doses of puerarin or the positive drug fluvastatin. Through classical HE staining of mouse aortic roots slices (Figure 2A), it was seen that the model control group has typical AS lesion characteristics, with obvious thickening of the vascular intima, lipid deposition, and massive formation of fibrous plaques. For the puerarin-treated group, as the dosage of puerarin increased, the vascular lesions were gradually reduced compared with the model control group, and the plaque area was also remarkably reduced. When the dosage of puerarin was up to 200 μM, the degree of plaque reduction was nearly the same as that of the fluvastatin group. The HE scores of the 200 μM puerarin group and fluvastatin group were significantly lower than the control group (*P* < 0.01 or *P* < 0.001). Furthermore, we observed the expression level of α-SMA in the plaque tissues of the aortic vascular wall of each group through the IHC method. The results showed that there were a large number of brown-yellow particles in the cytoplasm of the vascular wall and AS plaque surface of the model group. With the increase of the dosage in the puerarin group, the brown-yellow particles in the cytoplasm of the blood vessel wall and AS plaque surface decreased, and the fluvastatin group had the least brown-yellow particles (Figure 2B). Moreover, Oil Red O staining results showed that there were a large number of lipid droplets in the aortic roots of the model group. After treatment with puerarin, the lipid droplets in the plaque gradually decreased as the concentration increased, and the stained lipid droplets in the 200 μM puerarin group and fluvastatin group were similar (Figure 2C). To evaluate whether puerarin alleviates inflammation in AS mice, we used ELISA to detect the levels of inflammatory factors IL-6 and IL-8 in the serum (Figure 2D, left) and tissues (Figure 2D, right) of mice. Compared with the model group, the levels of IL-6 and IL-8 of the mice fed with low, medium, and high doses of the puerarin group and the fluvastatin group were significantly reduced (*P* < 0.05, *P* < 0.01 or *P* < 0.001). The above results indicated that puerarin effectively relieved the symptoms of AS mice, decreased the size of vascular plaques, reduced the expression of α-SMA, and alleviated the inflammatory response.

### Effects of puerarin on ox-LDL-induced hVSMC cells

**Figure 3.**
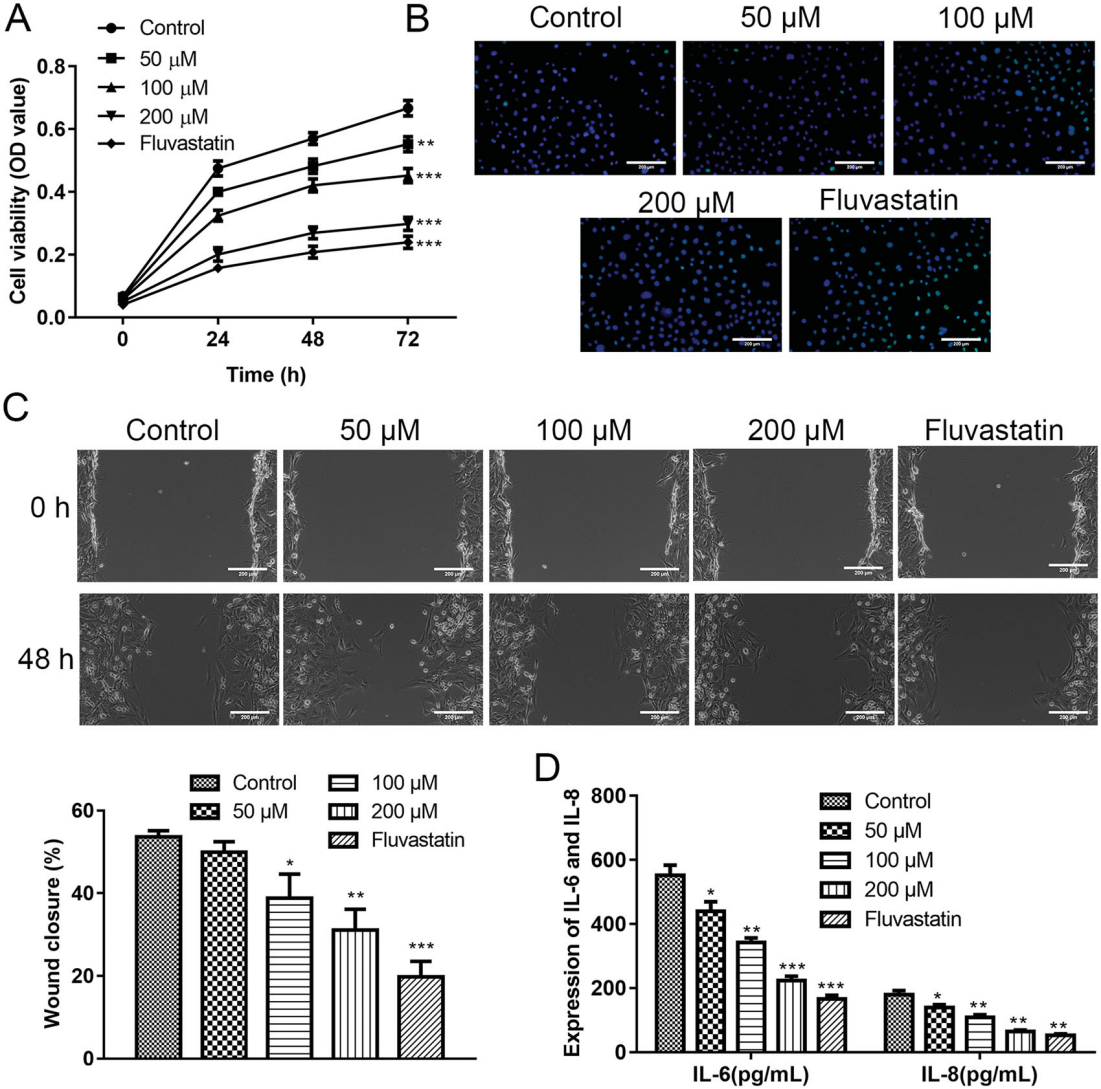
Puerarin inhibits the growth of ox-LDL-induced hVSMC cells. (A) Cell viability of ox-LDL-induced hVSMC cells in the control group, 50, 100, 200 μM puerarin-treated groups and fluvastatin-treated group. (B) Cell apoptosis of ox-LDL-induced hVSMC cells in 5 groups was detected by TUNEL staining. (C) The cell migration ability of ox-LDL-induced hVSMC cells in five groups was assessed by wound healing assay. Scale bar = 100 μm. (D) Expression of pro-inflammatory factors IL-6 and IL-8 of ox-LDL-induced hVSMC cells in five groups. *P < 0.05, **P < 0.01, ***P < 0.001.

Encouraged by the animal experiment, we speculated that puerarin also alleviated human AS. Therefore, we intended to investigate the function of puerarin on ox-LDL-induced hVSMC cells *in vitro* first. First, the function of puerarin on the proliferation of ox-LDL-induced hVSMC cells was assessed through the CCK-8 experiment. As exhibited in Figure 3A, the ox-LDL-induced hVSMC cells in the control group had the strongest proliferation ability. After treatment with 50, 100, and 200 μM puerarin, the cell viability of hVSMC cells after being induced with ox-LDL was weakened (50 μM group: *P* < 0.01, 100/200 μM and fluvastatin group: *P* < 0.001). The cell viability of the 200 μM puerarin group and the positive drug fluvastatin group was in substantial agreement, which indicates that puerarin significantly inhibits the proliferation of hVSMC cells under ox-LDL conditions. We detected the apoptosis of ox-LDL-induced hVSMC cells by TUNEL staining, it was seen that with the increase in the dosage of puerarin, the apoptosis of hVSMC cells gradually increased under ox-LDL conditions (Figure 3B). Moreover, the wound healing results showed that 100 and 200 μM puerarin significantly inhibits the migration of ox-LDL-induced hVSMC cells (*P* < 0.01), and the inhibitory effect of 50 μM puerarin on the migration of ox-LDL-induced hVSMC cell was relatively weak (*P* < 0.05) (Figure 3C). By detecting the content of inflammatory cytokines IL-6 and IL-8 in the supernatant of ox-LDL-induced hVSMC cells, we also found that puerarin reduced the inflammatory response (*P* < 0.05, *P* < 0.01 or *P* < 0.001) (Figure 3D).

### Puerarin exerts its effects through miR-29b-3p

**Figure 4.**
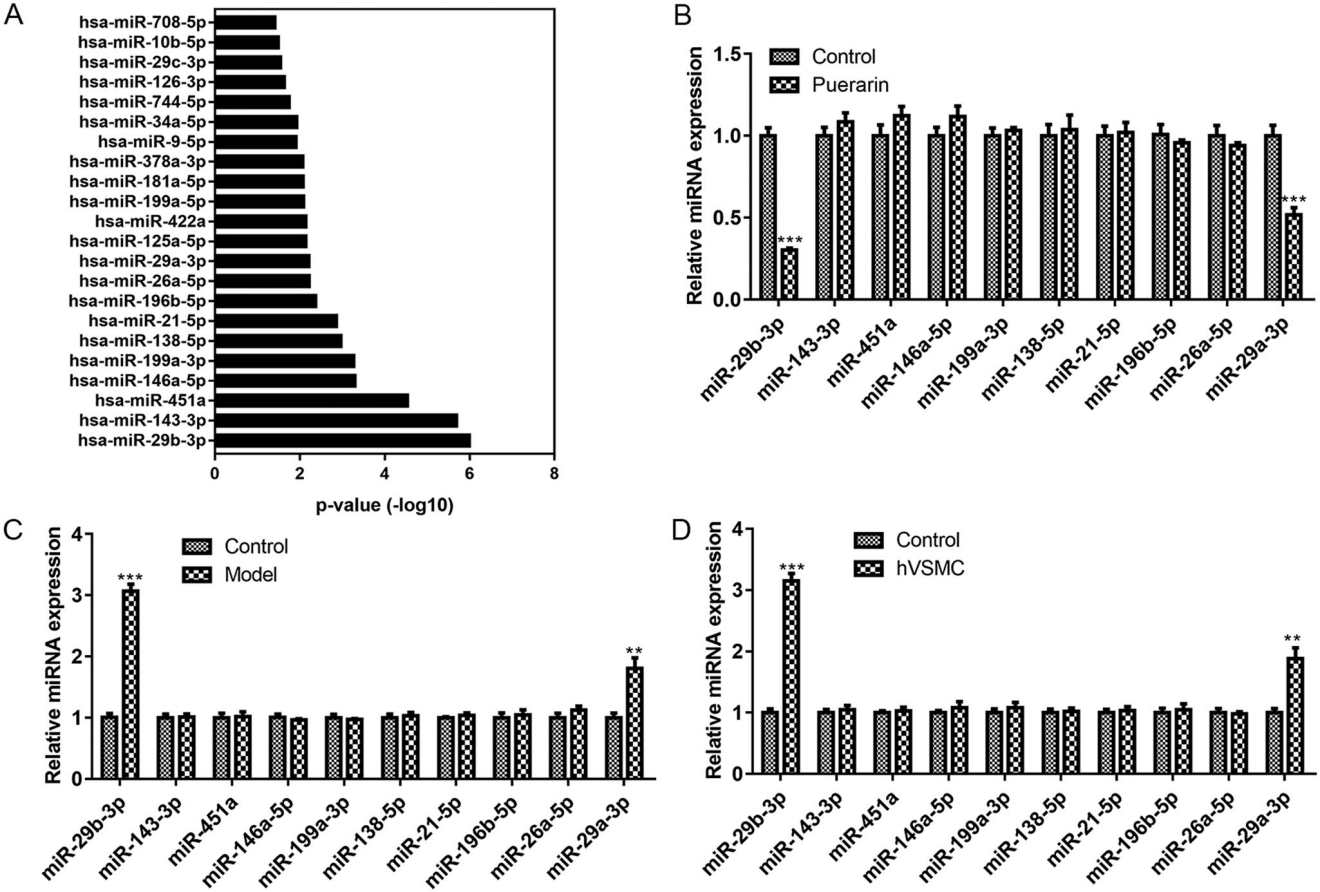
MiR-29b-3p is the target of puerarin. (A) GO function analysis was performed on the targets that puerarin intersects with atherosclerosis to screen out the target miRNAs of puerarin and ranked them in ascending mode of *P* value. (B) Changes in the expression of the ten screened miRNA in ox-LDL-induced hVSMC cells treated with puerarin. (C and D) The expression level of the ten screened miRNAs in the atherosclerosis mouse model (C) and ox-LDL-induced hVSMC cells (D).

To further investigate the molecular mechanism of puerarin in the therapy of AS, we first performed GO function analysis on the 70 targets that puerarin intersects with AS to screen out the target miRNAs and ranked them in ascending order of *P*-value. As seen in Figure 4A, miR-29b-3p was the most relevant to AS among all miRNAs screened. Then we analysed the effect of puerarin on the expression of the top ten miRNAs, and we found that after treatment with 200 μM puerarin, the expression of miR-29b-3p and miR-29a-3p in ox-LDL-induced hVSMC cells decreased significantly (*P* < 0.001) (Figure 4B). Subsequently, we verified the expression of these ten miRNAs in the AS mouse model and ox-LDL-induced hVSMC cells, respectively. The results exhibited that the expression levels of miR-29b-3p and miR-29a-3p were increased remarkably in AS model mice and ox-LDL-induced hVSMC cells (*P* < 0.001 and *P* < 0.01) (Figure 4C,D). Based on the above screening and validation results, we confirmed that puerarin exerts its therapeutic effect in AS through miR-29b-3p.

### Overexpression of miR-29b-3p reverses the effect of puerarin on hVSMC

**Figure 5.**
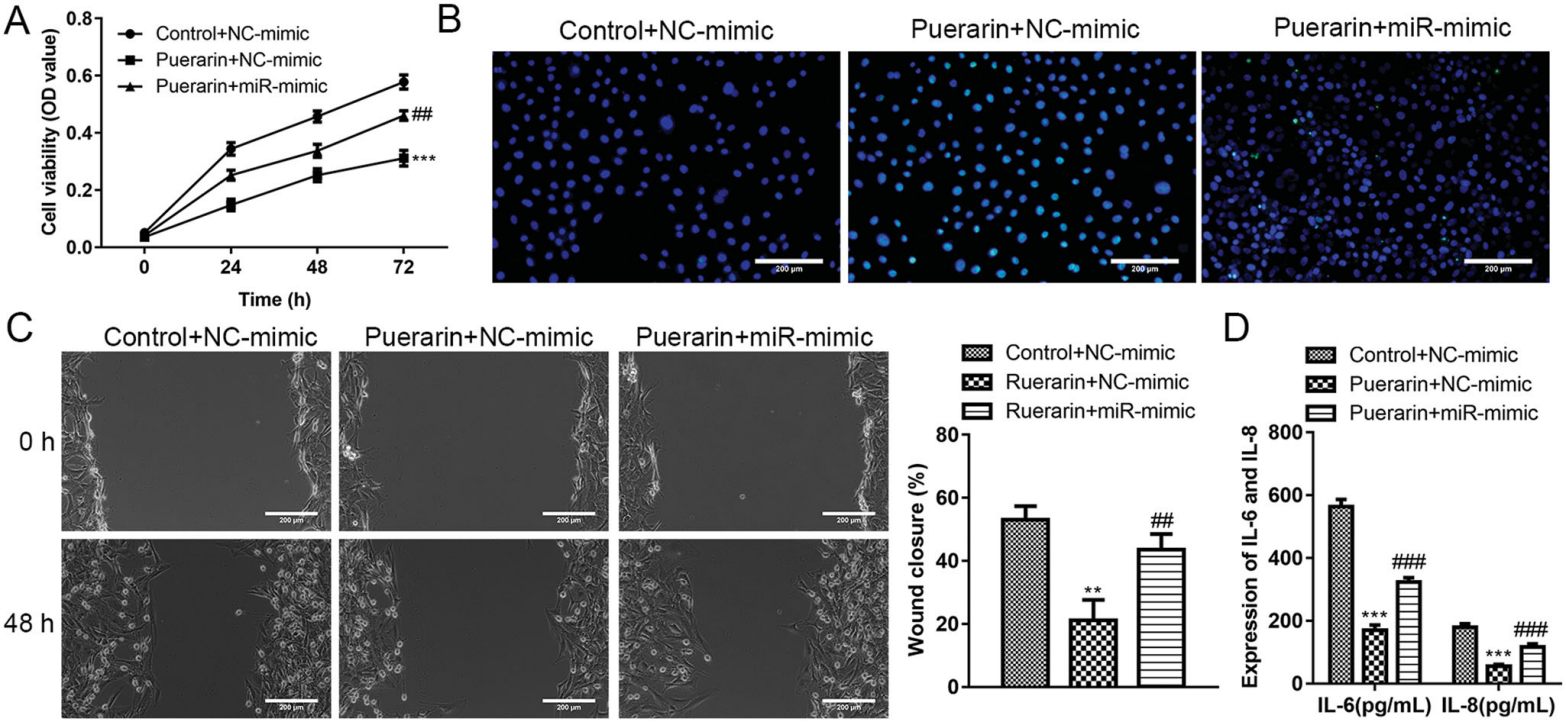
Overexpression of miR-29b-3p weakens the inhibition effect of puerarin on ox-LDL-induced hVSMC. (A) Cell viability of ox-LDL-induced hVSMC cells in the control + NC mimics group, puerarin + NC mimics group and puerarin + miR-29b-3p mimics group was detected at 24 h, 48 h, and 72 h. (B) Cell apoptosis of ox-LDL-induced hVSMC in the three groups was detected by TUNEL staining. (C) The cell migration ability of ox-LDL-induced hVSMC cells in the three groups was assessed by wound healing assay. Scale bar = 100 μm. (D) Expression of pro-inflammatory factors IL-6 and IL-8 of ox-LDL-induced hVSMC cells in the three groups. ^##^*P* < 0.01, ^###^*P* < 0.001, ***P < 0.001.

Next, we further verified that miR-29b-3p is the target of puerarin. As shown in Figure 5A–C, compared with the puerarin + NC mimics group, the overexpression of miR-29b-3p weakens the inhibition effect of puerarin on the proliferation and migration of ox-LDL-induced hVSMCs, and the promoting effect of puerarin on the apoptosis of ox-LDL-induced hVSMCs was also weakened. In addition, the inhibition effect of puerarin on the release of pro-inflammatory cytokines in hVSMC was reversed by the high expression of miR-29b-3p (Figure 5D).

### IGF1 is the potential target of miR-29b-3p

**Figure 6.**
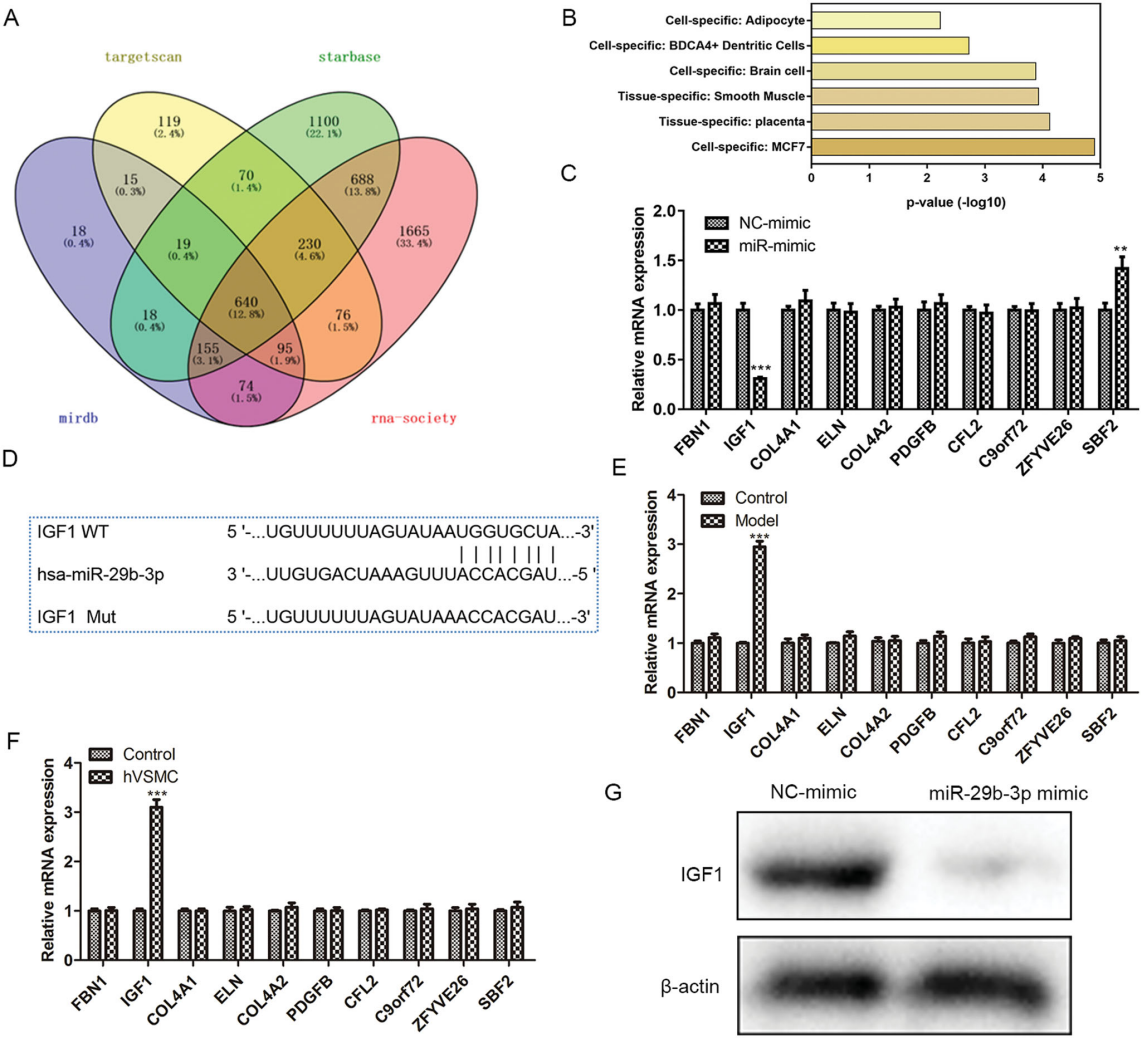
IGF-1 is the target of miR-29b-3p. (A) Target Scan, Starbase, miRDB, and RNA-society were used to predict the possible targets of miR-29b-3p. (B) PanGenBase was used to screen the target genes enriched in smooth muscle. (C) The expression level of the ten screened target genes was detected in ox-LDL-induced hVSMC cells over-expressing miR-29b-3p. (D) The binding site of the miR-29b-3p and the 3'UTR region of IGF-1. (E, F) The expression level of the 10 screened genes in the atherosclerosis mouse model (E) and (F) ox-LDL-induced hVSMC cells. (G) The binding of the miR-29b-3p and IGF-1 was verified by western blot. **P < 0.01, ***P < 0.001.

To make further efforts to explore how puerarin plays its role in the treatment of AS through miR-29b-3p, firstly, several different software was applied to forecast the downstream targets of miR-29b-3p (Figure 6A), and the PaGenBase database was applied to screen out a total of 10 target genes enriched in smooth muscle (Figure 6B). Subsequently, we overexpressed miR-29b-3p and detected the changes in the expression of these 10 target genes. We found that only the expression of IGF1 was markedly reduced when the miR-29b-3p was over-expressed (*P* < 0.001) (Figure 6C), indicating that IGF1 is the potential target of miR-29b-3p. Using the Targetscan software, we obtained the binding site of miR-29b-3p and IGF1 (Figure 6D). In addition, we used RT-qPCR to determine the expression level of the 10 target genes screened in AS mice and ox-LDL-induced hVSMC cells and found that only the level of the IGF1 gene was markedly increased (*P* < 0.001) (Figure 6E,F). Besides, Western Blot was conducted to verify the binding of miR-29b-3p and IGF1. After being transfected with miR-29b-3p mimic, the expression of IGF1 was reduced in hVSMC cells. The above results confirmed that IGF1 is the potential target of miR-29b-3p in AS.

### IGF1 and miR-29b-3p are negative correlation

**Figure 7.**
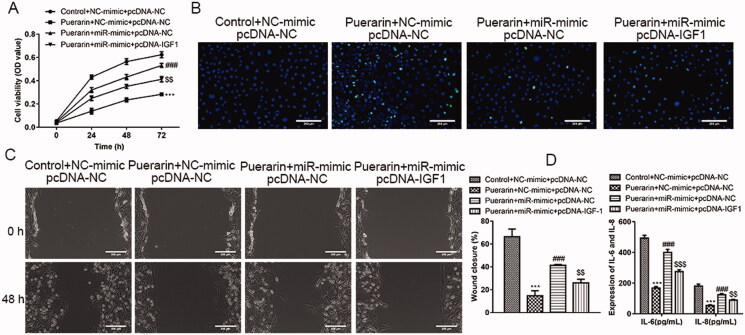
The function of miR-29b-3p is reversed by IGF1. The ox-LDL-induced hVSMC were divided into four groups and given different treatments: control + NC-mimics + pcDNA-NC, puerarin + NC-mimics + pcDNA-NC, puerarin + miR-mimics + pcDNA-NC and puerarin + miR-mimics + pcDNA-IGF1, the cell viability was determined by CCK-8 experiment (A), cell apoptosis was determined via TUNEL assay (B) and the cell migration was assessed by scratch test (C). The levels of inflammatory cytokines IL-6 and IL-8 secreted by cells were detected through ELISA (D). Scale bar = 100 μm. ^###^*P* < 0.01, ^$$^*P* < 0.05, ***P < 0.001.

We have confirmed that IGF1 is the potential target of miR-29b-3p, but the exact relationship between IGF1 and miR-29b-3p is still unclear. To this end, we explored their relationship through rescue experiments. Compared with the group that overexpresses miR-29b-3p and treated with puerarin, the cell viability of ox-LDL-induced hVSMC cells was reduced in the group that simultaneously overexpresses IGF1 and miR-29b-3p and added with puerarin, which successfully reversed the effect of miR-29b-3p overexpression (*P* < 0.01, Figure 7A). Similarly, in the TUNEL assay, compared with the control group, the apoptosis of ox-LDL-induced hVSMC cells in the group that was treated with puerarin was very obvious, while the apoptotic ox-LDL-induced hVSMC cells in the group that overexpressing miR-29b-3p alone and treated with puerarin was significantly reduced, while the apoptotic ox-LDL-induced hVSMC cells were increased slightly after simultaneously overexpressing IGF1 and miR-29b-3p and treated with puerarin (Figure 7B). The facilitation of cell migration caused by the overexpression of miR-29b-3p was also reversed (*P* < 0.01, Figure 7C). In addition, it was shown that after overexpression of IGF1, the number of inflammatory cytokines IL-6 and IL-8 secreted by the cells was reduced compared with the group that overexpresses miR-29b-3p alone (*P* < 0.01 or *P* < 0.001, Figure 7D). The above results indicate that there is a negative correlation between IGF1 and miR-29b-3p.

## Discussion

The occurrence and development of AS are strongly linked to inflammatory, oxidative stress and other processes, so it is particularly important to find effective prevention and treatment targets (Marchio et al. 2019). *P. lobata* is one of the traditional Chinese medicines with a wide range of clinical applications, and puerarin plays an indispensable role as one of its most important constituents. Here, we screened out puerarin as the active constituent of *P. lobata* in the treatment of AS, and the intersect targets of puerarin and AS were predicted. Studies have shown that puerarin can play an anti-AS function by lowering blood lipids, improving plaque size, and reducing the level of inflammatory factors. In addition, puerarin also inhibits the transmembrane transport of Na^+^ and K^+^ ions, reducing the vascular endothelial injury caused by inflammation, oxidative stress, and apoptosis (Bao et al. 2015). Recently, a study by Hu et al. (2020) indicated that puerarin reduces the expression of IL-6 and TNF-α in VSMC induced by oxidised low-density lipoprotein (ox-LDL), and significantly reduces MDA production and increases SOD activity. At the same time, puerarin can reduce the decline of VSMC cell viability caused by ox-LDL by inhibiting p38 MAPK and JNK pathways (Hu et al. 2020). The study by Ji et al. (2016) found that the therapeutic function of puerarin on AS is correlated to the reduction of inflammation and inhibition of NF-κB activation. In this study, by establishing AS mouse model and using hVSMC as a cell model, we demonstrated that puerarin can improve the symptoms of atherosclerosis in mice, inhibit the proliferation and migration of hVSMC, promote its apoptosis, and inhibit the levels of inflammatory cytokines IL-6 and IL-8 in mice and hVSMC.

MicroRNA is a kind of small non-coding RNA, which inhibits gene expression after transcription, and has been confirmed to be an important molecule involved in AS regulation. It has been found that multiple miRNAs are involved in the progression of AS (Giral et al. 2016). For example, miR-155 has been found to be a molecule involved in the inflammatory signalling pathway of AS (Bruen et al. 2019). Qiao et al. (2020) found that miR-210-3p reduces lipid accumulation in AS and inflammation response via inhibiting IGF2. Feng et al. (2018) found that miR-26a targets TRPC3 to inhibit the progression of AS. In addition, miR-21-3p, miR-92a, miR-126, miR-33, etc. have also been confirmed to be involved in the regulation of AS (Li et al. 2019; Zhu et al. 2019; Wang et al. 2019b). However, there is no relevant report on whether puerarin inhibits the occurrence and development of AS through any miRNA pathway. Here, we found that miR-29b-3p is decreased in AS mice and hVSMC, and miR-29b-3p is the target of puerarin to alleviate AS. Moreover, we confirmed that IGF1 is the target of miR-29b-3p.

Network pharmacology is a new subject that is widely used in the study of the complex mechanism of action of traditional Chinese medicines. It transforms the thinking framework of “one drug – one target – one disease” and uses an overall perspective to study the relationship between drug-target-disease. It has opened new ideas for the research of the pharmacology of Chinese medicine (Li and Zhang 2013; Boezio et al. 2017). Here, we used network pharmacology methods to screen and predict the constituents, diseases, and targets from multiple aspects. At the same time, we combined experiments to verify the screened targets, making the prediction results based on big data more realistic and credible, and providing a basis for the study of the molecular mechanism of puerarin treatment of AS. Next, we will screen more targets of puerarin for systematic study, so as to further clarify the mechanism of puerarin alleviating AS.

## Conclusions

In this study we confirmed that puerarin is the active constituent of *P. lobata*, it can alleviate AS by inhibiting the proliferation and migration of hVSMC, promoting apoptosis and inhibiting inflammation. In addition, we proved that puerarin alleviates AS through the miR-29b-3p/IGF1 pathway. This study illustrated that puerarin has a positive effect in alleviating atherosclerosis, and it also provided a theoretical basis and experimental basis for the research and application of puerarin in the treatment of atherosclerosis. Meanwhile, it also provides a new strategy for the treatment of atherosclerosis.

## Data Availability

The datasets used and/or analysed during the current study are available from the corresponding author on reasonable request.

## Appendix

Access of the database or software involved in this manuscript:

TCMSP: http://tcmspw.com/tcmsp.php

CTD: http://ctdbase.org/

Venn diagram: http://bioinfogp.cnb.csic.es/tools/venny/index.html

DAVID: https://david.ncifcrf.gov/home.jsp

Target Scan: http://www.targetscan.org/vert_72/

miRDB: http://mirdb.org/

Starbase: http://starbase.sysu.edu.cn/

RNA-society: http://www.rna-society.org/raid/

PaGenBase database: http://bioinf.xmu.edu.cn/PaGenBase/index.jsp
